# The impact of lowering the study design significance threshold to 0.005 on sample size in randomized cancer clinical trials

**DOI:** 10.1017/cts.2023.699

**Published:** 2023-12-18

**Authors:** Tiffany H. Leung, James C. Ho, Xiaofei Wang, Wendy W. Lam, Herbert H. Pang

**Affiliations:** 1 Department of Medicine, Li Ka Shing Faculty of Medicine, The University of Hong Kong, Hong Kong, China; 2 Department of Biostatistics and Bioinformatics, School of Medicine, Duke University, Durham, NC, USA; 3 School of Public Health, Li Ka Shing Faculty of Medicine, The University of Hong Kong, Hong Kong, China

**Keywords:** *P*-value, threshold, trial design, clinical trial, sample size

## Abstract

The proposal of improving reproducibility by lowering the significance threshold to 0.005 has been discussed, but the impact on conducting clinical trials has yet to be examined from a study design perspective. The impact on sample size and study duration was investigated using design setups from 125 phase II studies published between 2015 and 2022. The impact was assessed using percent increase in sample size and additional years of accrual with the medians being 110.97% higher and 2.65 years longer respectively. The results indicated that this proposal causes additional financial burdens that reduce the efficiency of conducting clinical trials.

Lowering the significance threshold to 0.005 is one of the suggestions to improve producibility, given the low reproducibility of findings in biomedical research [1]. An investigation related to this issue was performed by Wayant *et al*. [2] by examining the previously published articles using the 0.005 threshold. The authors primarily focused on how the new p-value threshold would change the conclusion of the original research. As the significance testing level is set at study design stage, the impact of lowering the p-value threshold on the conduct of clinical trials has yet to be investigated. Another author pointed out that the false positive risk was observed among 22% of the examined paper by lowering the p-value threshold to 0.005 [3]. This study aims to examine how lowering the significance threshold can affect the sample size and study duration by simulating the scenario using recently published articles.

Considering the relatively small sample size in phase I trials and the sophisticated study design with unpublished interim analysis in phase III trials, phase II clinical trials are believed to be more suitable for our study. Given the number of cancer trials being conducted each year is larger than those of other diseases, such as diabetes or cardiovascular diseases [4], and that the findings based on cancer clinical trials should be applicable to other diseases, this study was conducted using cancer trials. A literature search of phase II cancer trials targeting research works published between 2015 and 2022 in *JAMA Oncology*, *Journal of Clinical Oncology*, and *Lancet Oncology* was conducted using PubMed (Fig. 1) using the following search term: ((((cancer) AND randomized) AND (phase II OR phase 2)) AND (((“JAMA oncology”[Journal]) OR “Journal of clinical oncology: official journal of the American Society of Clinical Oncology”[Journal]) OR “The Lancet. Oncology”[Journal])) AND ((“2015/01/01”[Date - Publication] : “2022/01/31”[Date - Publication])). In total, 593 potentially relevant published articles were screened after the initial search, and 321 published articles were omitted because their study designs did not match our criteria.

**Figure 1.**
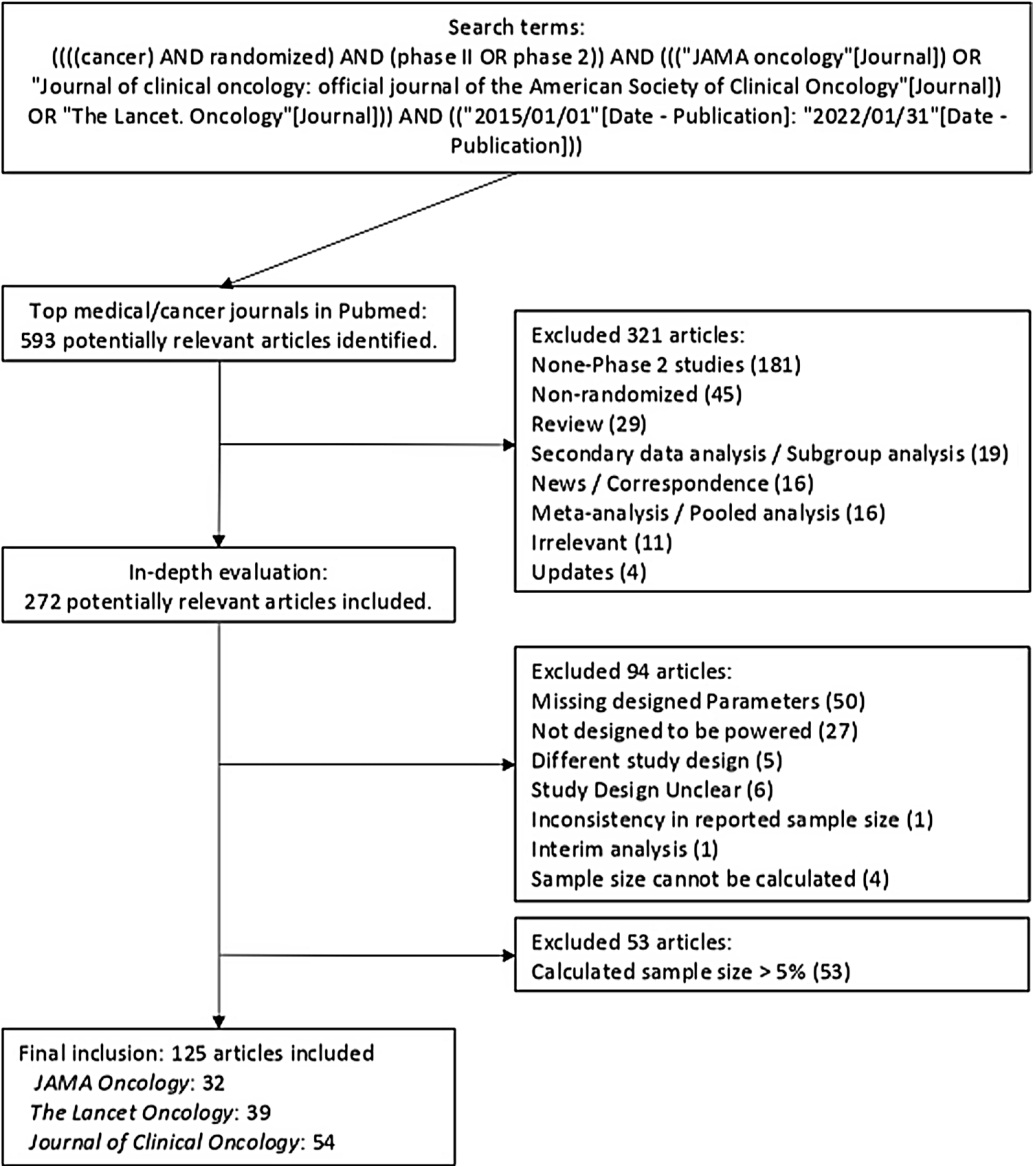
Process of literature selection.

Study design parameters, such as power, type I error, hazard ratio, accrual duration, follow-up duration, survival proportion, and survival time were collected according to the study design presented in the published articles. The sample size was calculated using the provided parameters to see if the original estimated sample size could be obtained. Among the 271 investigated articles, 50 of them were excluded since the details for calculating sample size were neither reported in the manuscript nor the supplement materials such as protocols and appendices. Consequently, 53 published articles with a greater than 5% difference between the original estimated sample size and our calculated result were also excluded. The purpose of excluding these studies was to avoid the situation in which the impact of lowering the significance threshold, such as percent increase in sample size and additional years of accrual, cannot be accurately calculated. The exclusion was performed and validated by different authors. The number of published articles included in study has amounted to 125 eventually, with 32, 54, and 39 published articles from *JAMA Oncology*, *Journal of Clinical Oncology*, and *Lancet Oncology,* respectively.

The sample sizes for the remaining articles were re-calculated using a type I error of 0.005 while keeping other parameters the same to obtain the sample size needed. Percent increase in sample size was defined as the difference between the actual recruited sample size and the re-calculated sample size divided by the actual recruited sample size. Furthermore, assuming that the accrual rates are the same before and after lowering the type I error threshold, the study duration was calculated by dividing the sample sizes by the accrual rates.

The characteristics of the selected 125 published articles are outlined in Table 1. They mainly cover different types of cancers: 21 (17%) were related to gastrointestinal cancer, and 20 (16%) to both genitourinary cancer and breast cancer.

**Table 1.** Characteristics of the included trials

Characteristics	No. (%) of papers (*n* = 125)
**Cancer type**	
Gastrointestinal	21 (17)
Breast	20 (16)
Genitourinary	20 (16)
Lung	16 (13)
Female reproductive organs	13 (10)
Hematological	11 (9)
Others	23 (18)
Unknown	1 (1)
**Type I error (design)**	
<0.05	6 (5)
0.05	51 (41)
0.1	38 (30)
>0.1	30 (24)
**Power (design)**	
<0.8	4 (3)
0.80–0.84	78 (62)
0.85–0.89	11 (9)
> = 0.9	32 (26)
**Primary endpoint**	
Progression-free survival	84 (67)
Overall survival	14 (11)
Objective response rate	7 (6)
Disease-free survival; Recurrence-free survival; Event-free survival	5 (4)
Complete response rate	5 (4)
Co-primary endpoints	5 (4)
Others	5 (4)

The percent increase in sample size is provided in Figure 2a. It ranged from 2.26% to 397.95% with a median of 110.97% and an IQR of 95.96%. Figure 2b illustrates the required additional years in study duration. The added years required ranged from 0.01 years to 17.02 years. The median and the IQR of the additional years of accrual were 2.65 years and 2.92 years respectively. To understand the levels of impact using different significance thresholds, p-values were also adjusted to 0.05, 0.025, and 0.01. The results are summarized in Supplementary Figure 1. In addition, a validation of our findings was conducted using articles published between 2015 and 2022 in NEJM. The results were consistent with our findings with an increase in sample size and trial duration being observed. This indicates that only targeting the three proposed should not severely affect the generalizability of our findings.

**Figure 2.**
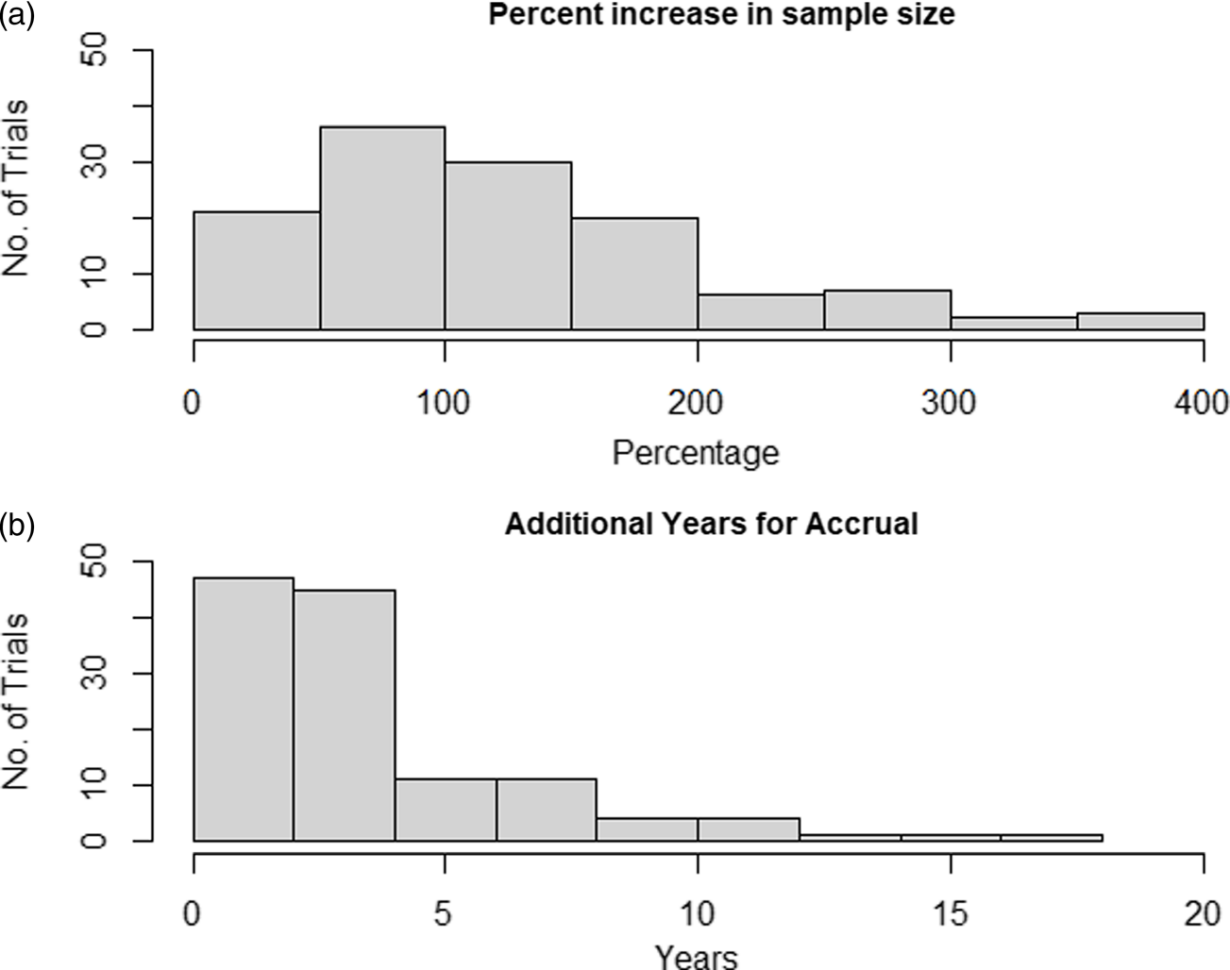
Distribution of percent increase in sample size and additional years of accrual after lowering p-value threshold.

Increased sample size and years of accrual have impacts on finances and the duration of trials. The impact of lowering the significance threshold is particularly important since more than half of the trials will double their expected sample size. Although the increase in sample size due to lowering the significance threshold was already discussed, our study found that 67.2% of the included articles required more than 70% of the original sample size, indicating that the impact of lowering the significance threshold may be underestimated in previous discussions [5]. Extra administrative work and high treatment cost due to longer trial duration with larger sample size will add burden to running trials. This can be further contemplated that the average cost of phase II oncology trials between 2004 and 2012 was USD 11.2 million, of which 93% of the trial cost was attributed to sample size and trial duration [6]. If the significance threshold were lowered, the trial cost would become USD 18.4 million to USD 29.1 million (the first and the third quartile of the percent increase being applied). This is based on the assumption that the percentage of trial cost attributed to sample size and trial duration remains 93% regardless of lowering the significance threshold or not. For the larger phase III trials, the average total cost of USD 22.1 million between 2004 and 2012 might grow beyond USD 100 million if the significance threshold is lowered. This change is due to the per-patient basis of multiple trial cost components rather than other factors (e.g. Institutional Review Board (IRB) application and data management). Besides treatment costs, the workloads for site monitoring of trials, which are usually conducted every four to eight weeks, will similarly expand [6]. Furthermore, the large number of required samples can result in study termination because of slow accrual. Unnecessarily large studies led by lowered significance threshold can also result in statistically significant findings but are less clinically meaningful, such as an extra gain of 2.1 months in overall survival [7]. Although lowering the significance threshold had been proposed to balance the impact of growth in sample size, this proposal is less ideal because it increases the chance of incorrectly concluding a promising therapy as insignificant [8].

Considering the long trial duration and high cost, lowering the significance threshold at the study design may not be a reasonable solution to ensure reproducibility. Alternatively, tackling other factors such as poor research practice and publication bias may be more feasible for enhancing producibility. Publication bias can be found in many disciplines as authors and journals have a higher preference for significant results [9]. One possible solution is to further encourage the publication of non-significant results [10]. Meta-analysis is another solution since it generates a precise estimate of trial effect based on the larger sample size pooling from different trials with similar research interests [11]. The US National Institutes of Health also advocates for improved trial practice with better experimental design and research practice with more clearly stated details (e.g. power, follow-up duration) to help improve reproducibility [12]. As for study design, one may consider Bayesian approaches in trial designs. For example, calculating the reproducibility possibility and adjusting the designed sample size accordingly to reach a desirable level [13]. Besides the Bayesian approach, the rapid growth in electronic patient record systems in recent years increases the practicality of applying real-world evidence in oncology trials, which can potentially improve the external validity of trials and reproducibility [14].

This is the first study examining the actual impact on trial duration after lowering the significance threshold at the study design stage, which helps understand the direct consequences on sample size and study duration. In addition, the presented approach can be applied to different phases of studies if the fundamental parameters and required information are provided. With this provision, we can estimate the level of impact on sample size and trial duration of both phase I and phase III studies.

This study is limited by the relatively few types of study designs. Published articles with more sophisticated study designs, such as noninferiority trial design and pick-the-winner design, are omitted owing to the lack of design information or great differences between the reported sample and our calculated result. In addition, interim analysis was not considered in our study calculations. This is supported by the results of our literature search as nearly all the identified studies did not terminate early due to interim analysis, if applicable. We believe that this is potentially due to the nature of the phase II study setting, which is less likely to be terminated early due to interim analysis. Another limitation is that this study was conducted based on the assumption that other factors, such as accrual rate and follow-up time for survival outcomes, remain the same, which is not likely in practice. However, considering that the accrual rate was calculated based on the empirical enrollment data from published study rather than based on the original study assumptions, we believe that our results are reasonable.

## Supporting information

Leung et al. supplementary material Leung et al. supplementary material

## Supplementary material

The supplementary material for this article can be found at https://doi.org/10.1017/cts.2023.699.
