# Supplementary material for: The impact of lowering the study design significance threshold to 0.005 on sample size in randomized cancer clinical trials
Source: J Clin Transl Sci. 2023 Dec 18;8(1):e9. doi: 10.1017/cts.2023.699 (PMC10877511; doi:10.1017/cts.2023.699)
Supplement: Leung et al. supplementary material [file S2059866123006994sup001.docx]

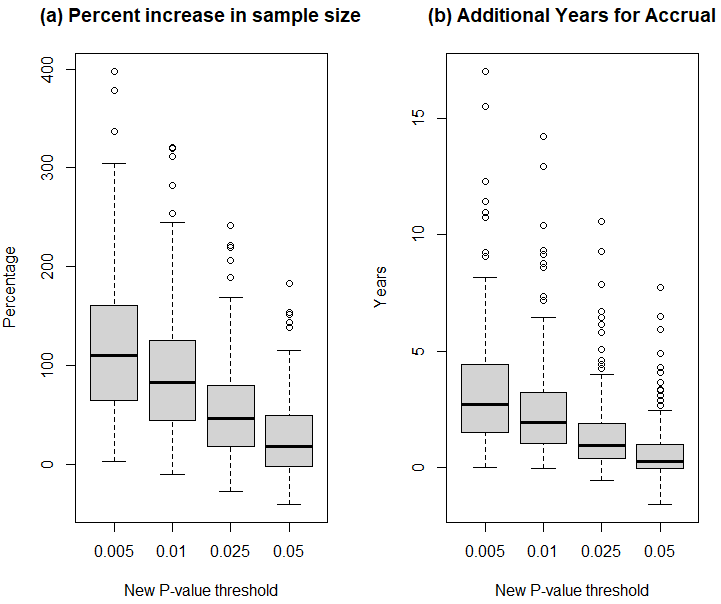


**Supplementary Figure 1.** Distribution of percent change in sample size and additional years of accrual of different significance thresholds
